# Robust neutralising activity and activation of neutrophil cytotoxic responses mediated by antibodies targeting the HTLV-1 envelope glycoprotein

**DOI:** 10.1186/1742-4690-8-S1-A170

**Published:** 2011-06-06

**Authors:** Chien-Wen Kuo, Lindsay Tulloch, David W Brighty

**Affiliations:** 1 The Biomedical Research Institute, College of Medicine Dentistry and Nursing, Ninewells Hospital, Dundee, DD1 9SY, UK

## 

Infection of human cells by Human T-cell leukaemia virus (HTLV-1) is mediated by the viral envelope glycoproteins. The gp46 surface glycoprotein binds to a cell surface receptors, including heparan sulphate proteoglycans, neuropilin-1, and Glut-1, allowing the transmembrane glycoprotein to initiate fusion of the viral and cellular membranes. The envelope glycoproteins are recognised by neutralising antibodies and cytotoxic T lymphocytes following a protective immune response, and therefore represent attractive components for an HTLV-1 vaccine. Here we begin to explore the immunological properties of recombinant SU as a potential subunit vaccine candidate. We have used a soluble recombinant gp46 fused to the Fc-region of human IgG (sRgp46-Fc) as an immunogen to vaccinate mice and generate monoclonal antibodies (mAbs) targeting SU. The recombinant SU protein is highly immunogenic, inducing high titre antibody responses to the immunogen and facilitating selection of hybridomas that secrete anti-SU mAbs. Many of these mAbs recognise envelope expressed by HTLV-1 infected cells and these antibodies strongly stimulate neutrophil-mediated cytotoxic responses. Moreover, several of the mAbs directly and robustly antagonise envelope-mediated fusion and neutralize pseudovirus infectivity. While antibodies targeting either the receptor-binding domain or C-terminal domain of SU exhibit anti-viral activity, the most potently neutralizing mAbs tend to recognise epitopes that cluster in the N-terminal receptor-binding domain of SU. Thus, our data demonstrate that recombinant forms of SU possess immunological features that are of direct utility to subunit vaccine design.

